# Supplementary material for: Adipose Tissue Mitochondrial Factors Profile after Dietary Bioactive Compound Weight Reduction Treatments in a Mice Obesity Model
Source: Int J Mol Sci. 2019 Nov 22;20(23):5870. doi: 10.3390/ijms20235870 (PMC6928597; doi:10.3390/ijms20235870)

Supplementary information

Adipose tissue mitochondrial factors profile after diet treatments

*Anna Cassanye, Meritxell Martín-Gari, Manuel Portero-Otin, José CE Serrano**

Department of Experimental Medicine, NUTREN-Nutrigenomics, University of Lleida

* Corresponding author: jceserrano@mex.udl.cat

Supplementary information contains:

- Supplementary Table S1 and S2
- Supplementary Figures S1 and S2

Table S1. *Dietary and nutritional composition of experimental diets*. Fish oil was purchased from Sigma-Aldrich (F8020 Lot#051M1861V), which contains 28.6% of Omega-3 fatty acids and 19.7% of palmitic acid. Soluble fibre, plantago ovata, was purchased from Laboratorios Normon, S.A. (Spain). Soy was purchased from a local supermarket (Vivesoy, Pascual-Spain), which has a nutritional composition of 19% fat, 27% carbohydrates (23% of sugars), 34% protein and 7.8% fibre.

**Ingredients (g/kg)**	**Normocaloric**	**High-fat**	**Normocaloric**			**High-fat**		
			Fish oil	Soluble fibre	Soy	Fish oil	Soluble fibre	Soy
**Casein**	245	245	245	245	235	245	245	235
**L-cysteine**	3.5	3.5	3.5	3.5	3.5	3.5	3.5	3.5
**Corn starch**	302	85	302	265	297	85	68	79
**Sucrose**	174	200	174	153	164	200	159	191
**Lard**	0	250	0	0	0	250	250	250
**Corn oil**	109	90	55	109	103	36	90	84
**Cellulose**	58	58	58	58	56	58	58	56
**AIN-93 Mineral mix**	43	43	43	43	43	43	43	43
**AIN-93 Vitamin mix**	19	19	19	19	19	19	19	19
**Calcium phosphate**	3.4	3.4	3.4	3.4	3.4	3.4	3.4	3.4
**Choline bitartrate**	3	3	3	3	3	3	3	3
**Bioactive compound**	-	-	54	58	33	54	58	33
**Water**	40.1	-	40.1	40.1	40.1	-	-	-
**Energy (kcal/g)**	3.88	5.19	3.88	3.65	3.88	5.19	4.96	5.19
**% Protein**	26	19	26	27	26	19	20	19
**% Fat**	25	59	25	26	25	59	62	59
**% Carbohydrates**	49	22	49	47	49	22	18	22

Table S2. *TaqMan probe assays for gene expression analysis.*

**Gene**	**Probe assay ID**
FASN	Mm00662319_m1
PPARG	Mm00440940_m1
IRS1	Mm01278327_m1
ADIPOQ	Mm00456425_m1
LEP	Mm00434759_m1
PLIN1	Mm00558672_m1
DGAT1	Mm00772290_m1
TNFALPHA	Mm00443258_m1
IL6	Mm00446190_m1
CYBA	Mm00514478_m1
TFAM	Mm00447485_m1
NRF1	Mm01135606_m1
18S	Mm03928990_g1

Figure S1. *Gene expression analysis in adipose tissue*. Results are presented as the expression ratio relative to 18S gene expression. Differences between groups were determined by one-way ANOVA and multiple comparisons were performed comparing the mean of each group with the mean of the Control normocaloric (a), Control High-Fat (b) and Treatment Normocaloric (c) diets. Correction for multiple comparisons was performed with Dunnett test and p-values below 0.05 (marked in bold) were considered as a significant difference between groups (* p<0.05, ** p<0.01, *** p<0.001)

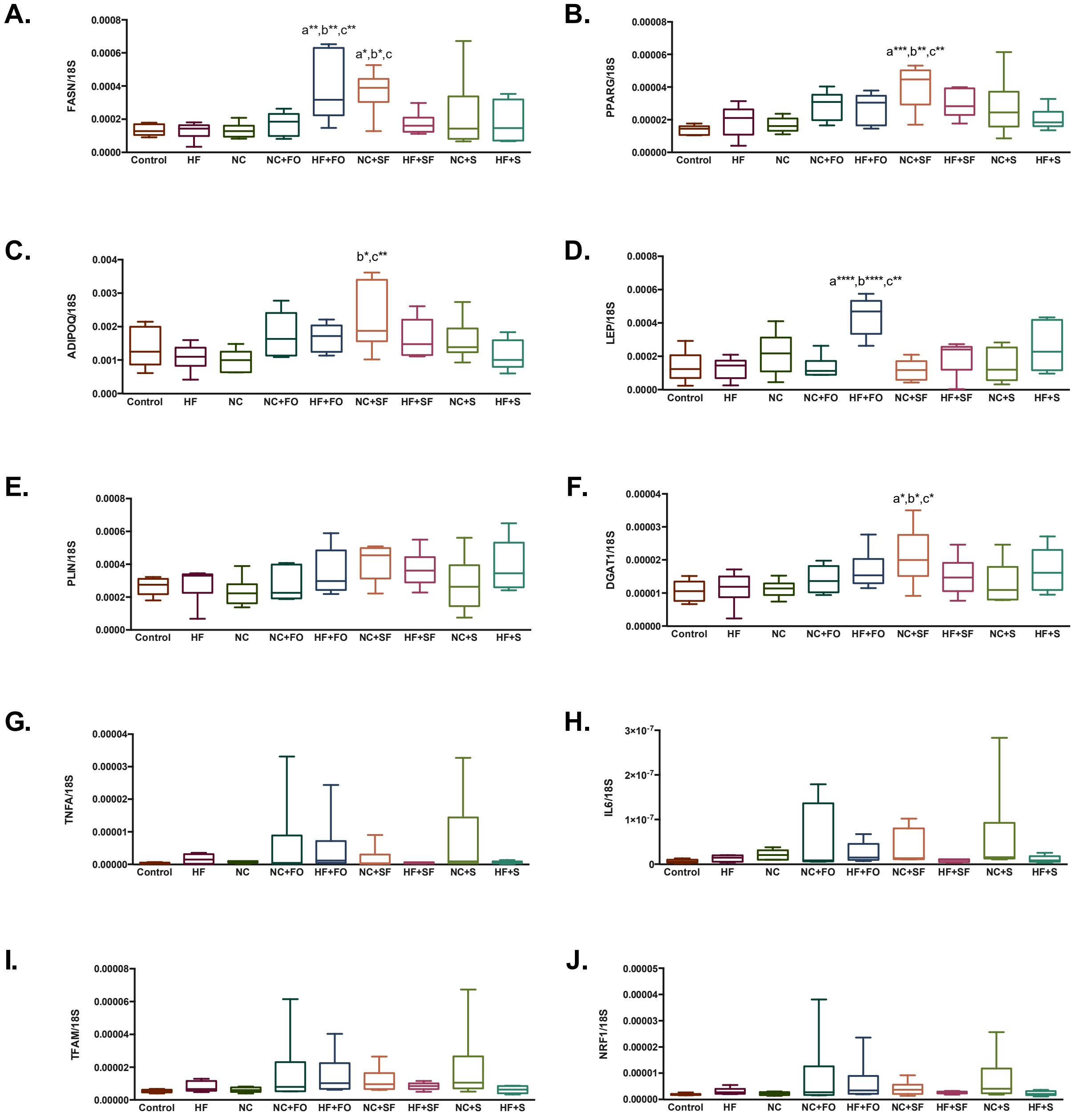

Figure S2. *Experimental procedure scheme*. The total duration of the study was 6 months. The first 4 months were dedicated to induce obesity through a high-fat diet. After that, mice were divided in 8 groups, where different treatments to induce metabolic changes were performed. A control group fed with a normocaloric diet was included through out the entire experimentation time.

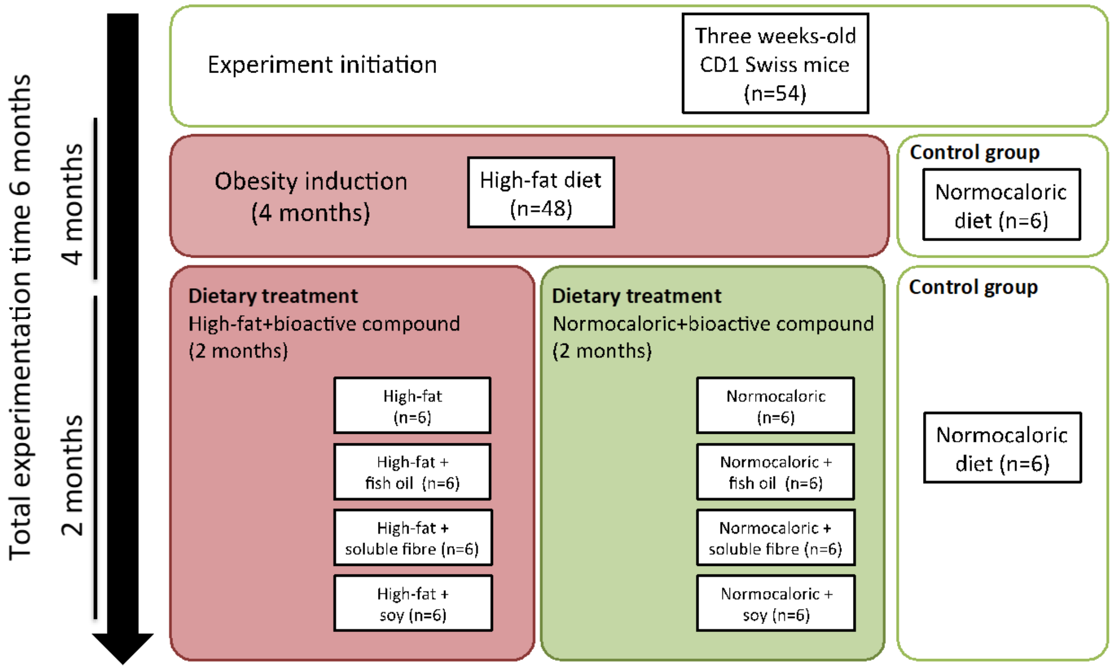

Supplement: Supplementary file 1 [file ijms-20-05870-s001.pdf]
